# Advancing the rights of persons with disabilities through global human rights frameworks

**DOI:** 10.1192/j.eurpsy.2026.10356

**Published:** 2026-07-31

**Authors:** K. Krysta

**Affiliations:** Department of Rehabilitation Psychiatry, Medical University of Silesia, Katowice, Poland

## Abstract

**Abstract:**

The global movement away from institutional models of care toward inclusive, community-based support for persons with disabilities represents one of the most significant transformations in contemporary social and mental health policy. This shift is grounded in international human rights standards, particularly the United Nations Convention on the Rights of Persons with Disabilities (CRPD), which emphasizes inherent dignity, individual autonomy, non-discrimination, equality of opportunity, and full participation in society. The CRPD challenges long-standing practices of segregation and substitute decision-making, calling instead for systems that enable independent living and meaningful inclusion in the community.

The abstract examines how these principles are translated into practice across mental health and social care systems worldwide. Drawing on assessments conducted using the World Health Organization’s QualityRights Toolkit, it identifies recurring structural and systemic barriers, including limited recognition of legal capacity, continued reliance on institutional care, inadequate access to community-based services, and insufficient individualized support for daily living and social participation. These challenges highlight the gap between formal policy commitments and lived experiences of persons with disabilities.

At the same time, the analysis presents evidence-based strategies and policy approaches that have demonstrated success in advancing deinstitutionalization and rights-based care. These include the development of supported decision-making frameworks, investment in community mental health services, cross-sector collaboration, and the active involvement of persons with disabilities in service design, monitoring, and evaluation. Particular attention is given to the role of legislative reform, workforce training, and cultural change in sustaining long-term transformation.

Through international case studies and comparative perspectives, the abstract offers practical insights for policymakers, clinicians, service providers, and advocates. It underscores the necessity of aligning mental health and disability services with human rights standards, not only as a legal obligation but as a foundation for ethical, effective, and person-centered care.

**Disclosure of Interest:**

None Declared

